# Ectopic hepatocellular carcinoma metastasizing to the brain with initial presentation as tumor stroke syndrome: a case report

**DOI:** 10.1093/bjrcr/uaag011

**Published:** 2026-04-11

**Authors:** Haiyang Li, Daoying Wang

**Affiliations:** Department of Urology, Gansu Provincial Hospital, Lanzhou, Gansu, 730000, China; Department of PET/CT Center, Gansu Provincial Hospital, Lanzhou, Gansu, 730000, China

**Keywords:** ectopic hepatocellular carcinoma, brain metastasis, tumor stroke syndrome, PET/CT, alpha-fetoprotein, FDG-avid mass, emergency surgery

## Abstract

Ectopic hepatocellular carcinoma (EHCC) with brain metastasis (BM) is exceptionally rare and carries a poor prognosis. We report a case initially presenting with tumor stroke syndrome, manifesting as sudden unconsciousness and unresponsiveness. Emergency intracranial hematoma evacuation was performed, and histopathology confirmed BM of hepatocellular carcinoma. Subsequent PET/CT localized the primary EHCC to the left lung lower lobe. This case underscores the critical role of radiological evaluation (CT/MRI for diagnosis/prognosis; PET/CT for primary lesion identification) in such scenarios. For patients with tumor stroke as the initial symptom, prompt suspicion of hemorrhagic malignant BM is essential, requiring emergent intervention and pathological confirmation. Notably, markedly elevated alpha-fetoprotein combined with extrahepatic FDG-avid masses on PET/CT strongly suggests EHCC.

## Introduction

Ectopic hepatocellular carcinoma (EHCC), defined as hepatocellular carcinoma (HCC) arising in extrahepatic organs without connection to the native liver,^1^^,^^2^ is an exceptionally rare entity with a reported incidence of 0.24%-0.47% for ectopic liver tissue.^3^ Among these cases, 7%-30% undergo malignant transformation.^4^ Common sites of occurrence include the gallbladder, followed by the thoracic cavity, perihepatic parenchyma, pancreas, retroperitoneum, spleen, and adrenal gland.^5–7^ Preoperative diagnosis remains challenging due to nonspecific imaging features. Notably, EHCC metastasizing to the brain and presenting as an acute stroke syndrome has not been previously documented. We report a unique case of EHCC originating in the left lower lobe of the lung, which initially presented with hemorrhagic brain metastasis (BM) and severe neurological deficits. Histopathological examination of the evacuated hematoma confirmed HCC. This report aims to enhance clinical awareness of this rare condition and provide insights into the management of EHCC-related stroke syndromes, thereby contributing to future research.

### Clinical presentation

A 55-year-old male presented with acute unconsciousness lasting 5 h, without systemic symptoms (fever, vomiting, or seizures). Neurological examination demonstrated somnolence, asymmetric pupils (right 4 mm, left 2 mm) with intact light reflexes, symmetric cranial nerves, grade 2 limb muscle strength, and bilateral Babinski positivity. No significant medical history (hypertension/diabetes/malignancy) was noted.

### Investigations and imaging findings

**Figure 1:**
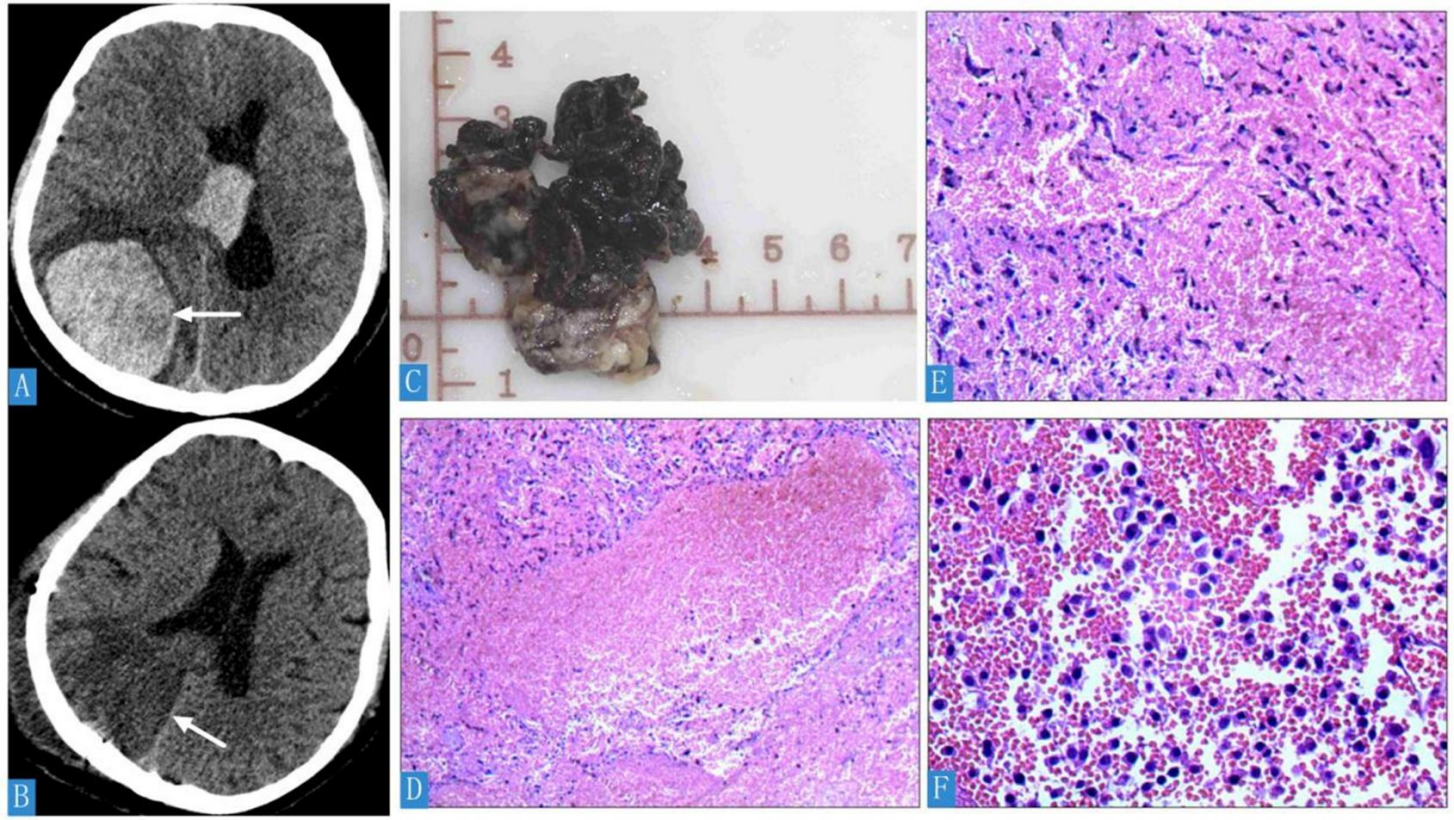
Neuroimaging and histopathological findings. (A) Non-contrast cranial CT demonstrating right parieto-occipital hemorrhage (estimated volume, 70 mL) with intraventricular extension (arrow). (B) Postoperative CT showing right cerebral hemisphere edema with focal involvement of the temporo-parieto-occipital region (arrow). (C) Resected brain tissue specimen (tumor stroke). (D-F) Histopathology of hemorrhagic tissue (H&E staining). (D) Densely arranged clusters of small degenerated cells (H&E, ×100). (E) Tumor cells expressing hepatocellular carcinoma markers (H&E, ×100). (F) High-power view of atypical cellular morphology (H&E, ×400).

**Figure 2:**
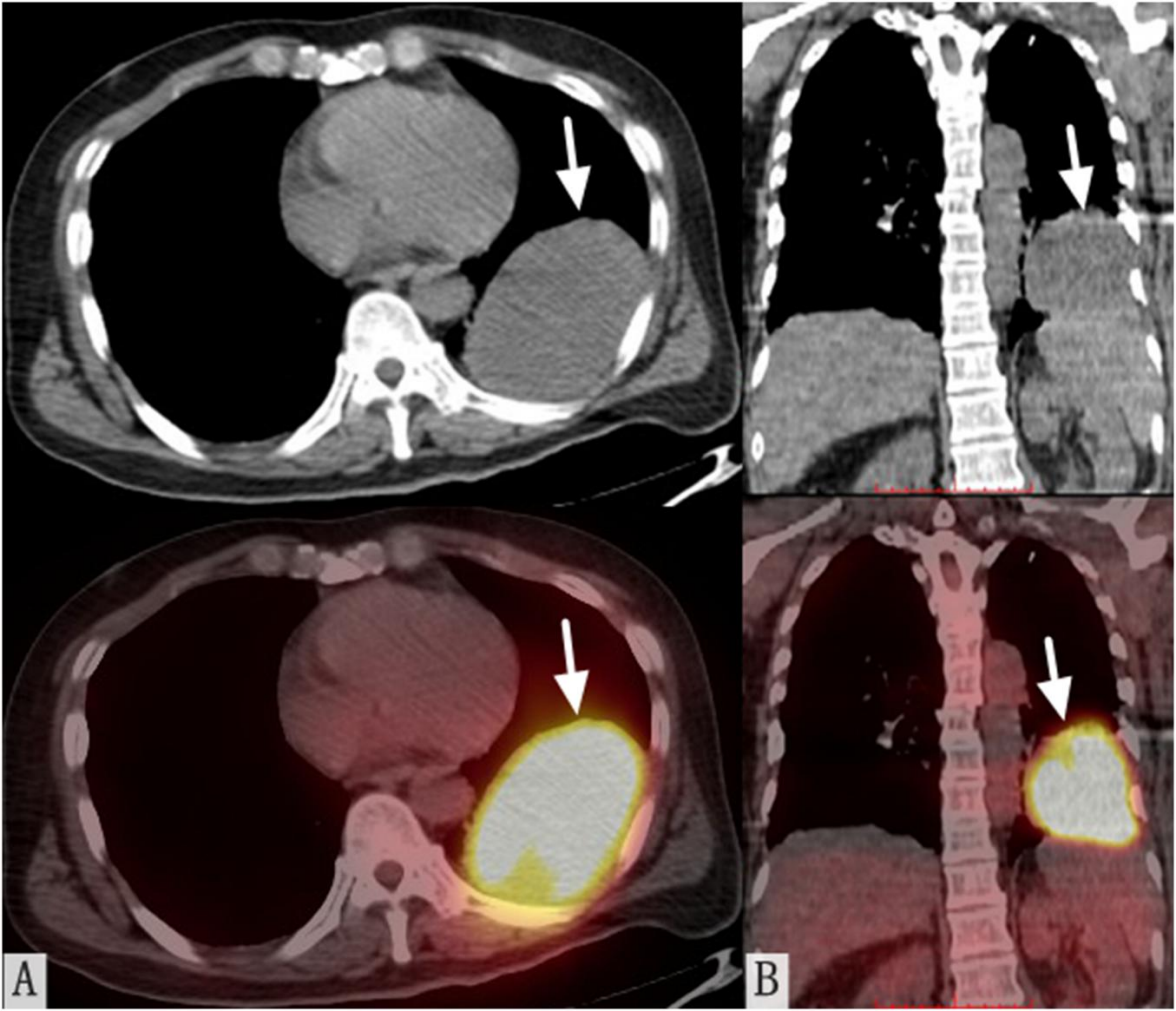
^18^F-FDG PET/CT findings. (A and B) Axial (A) and coronal (B) fused PET/CT images demonstrating an 8.7 × 6.4 × 8.2 cm FDG-avid soft tissue mass in the left lower lung lobe (arrows). No hepatic lesions or abnormal metabolic activity were observed.

Emergency head CT revealed a 5.9 × 5.0 cm hyperdense mass in the right parieto-occipital lobe with intraventricular hemorrhage (70 mL volume), midline shift, and perilesional edema (Figure 1A). Histopathology of resected brain tissue showed degenerated tumor cell clusters; immunohistochemistry supported hepatocellular origin (Arg-1[+], CK19[weak+], GPC-3[+], CKP[−], EMA[−], Vimentin[−], CK7[−], TTF-1[−], NapsinA[−], CEA[−]; Ki-67 60%) (Figure 1C-F). Serum AFP exceeded 2000 ng/mL. Systemic imaging identified an 8.8 × 6.0 cm left lower lung mass on CT, with PET/CT confirming intense FDG uptake (SUV_max_ 24.59) without hepatic involvement (Figure 2A and B).

### Differential diagnosis

The diagnostic workup prioritized the exclusion of AFP-producing pulmonary hepatoid adenocarcinoma (HAC), a known mimic of HCC. The immunohistochemical findings, however, unequivocally favored hepatocellular origin. The tumor demonstrated a classic HCC phenotype: diffuse Arg-1 positivity confirming hepatocellular differentiation, and a complete lack of CK7, which is aberrant in HAC. This, combined with negativity for pulmonary markers (TTF-1, Napsin A), served to exclude a primary pulmonary adenocarcinoma variant. The observed FDG avidity aligns with the tumor’s high-grade nature, supported by the elevated Ki-67 proliferation index.

### Treatment, outcome, and follow-up

**Figure 3:**
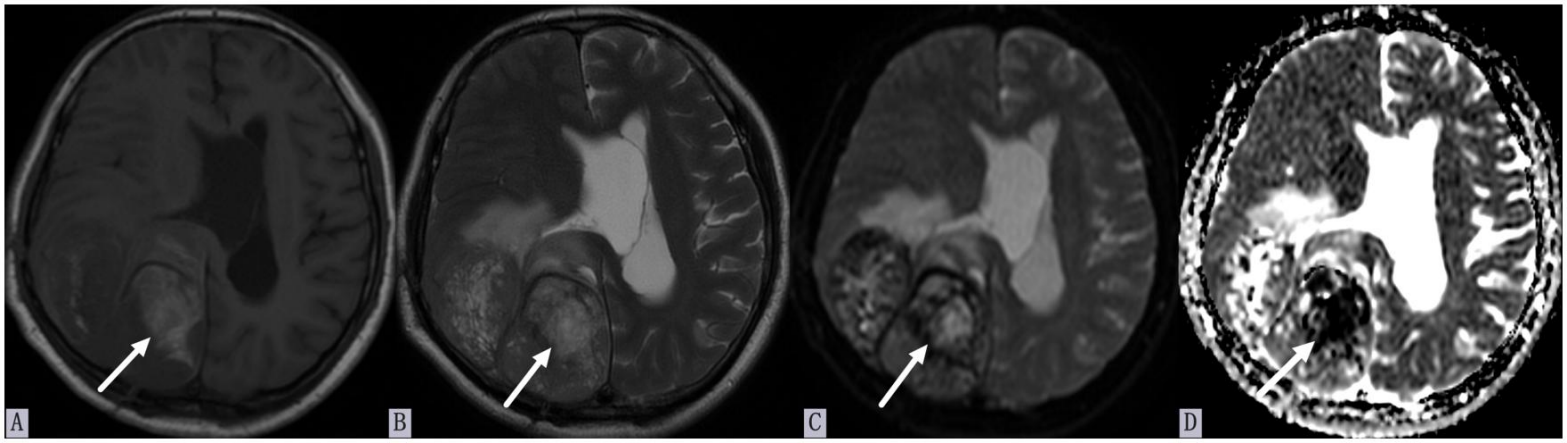
Brain MRI findings at 2-month follow-up. (A) Axial T1WI showing a well-defined right temporo-parieto-occipital mass with intrinsic T1 hyperintensity (arrow). (B) Axial T2WI demonstrating T2 hyperintensity within the mass (arrow). (C) DWI revealing restricted diffusion in the lesion (arrow). (D) ADC map confirming corresponding hypointensity (arrow).

Emergency craniotomy evacuated the hematoma. Multidisciplinary consensus diagnosed EHCC (lung primary) with hemorrhagic BM. Postoperative CT showed residual right hemispheric edema (Figure 1B). At discharge, neurological function significantly improved (consciousness clear, limb strength grade 5), though right ptosis and bilateral Babinski signs persisted. Two-month follow-up MRI demonstrated a persistent hemorrhagic lesion in the right occipital lobe with mass effect and edema (Figure 3A-D). Biopsy of the lung lesion and further medical intervention were not performed due to the patient’s acute presentation and severe neurological deterioration.

## Discussion

Brain metastasis, a devastating neurological complication affecting 20%-40% of patients with advanced cancer,^8^ is uncommon in HCC, with an incidence of only 0.2%-2%.^9^^,^^10^ However, 33.9%-70% of these metastases are complicated by hemorrhage, a condition known as “tumor stroke,” which is attributable to fragile tumor neovasculature, vascular invasion, or coagulopathy.^11^^,^^12^

We report a case of EHCC originating in the left lower lung lobe, which initially manifested as acute hemorrhagic BM mimicking a stroke syndrome. This case is distinctive in that it combines two highly uncommon features: a primary pulmonary origin and a debut as tumor stroke. To our knowledge, this represents the first documented instance of EHCC exhibiting this specific clinico-radiological evolution. Our report not only expands the known clinical spectrum of EHCC but also highlights the importance of considering EHCC in the differential diagnosis of unexplained intracranial hemorrhage accompanied by a pulmonary mass.

The patient presented with acute consciousness disturbance, a symptom attributed to the hemorrhagic BM rather than a direct effect of the primary EHCC. Initial imaging revealed a left-sided intracranial hemorrhage and a solitary nodule in the left lower lung. Subsequent ^18^F-FDG PET/CT confirmed hypermetabolism in the pulmonary lesion and ruled out other primary sites. A markedly elevated serum alpha-fetoprotein (AFP) level provided key laboratory support for a tumor of hepatocellular origin.

The diagnostic evaluation centered on distinguishing AFP-producing HAC of pulmonary origin—a well-recognized extrahepatic malignancy that synthesizes AFP and closely simulates HCC in morphology and clinical presentation—from primary HCC or EHCC. Prior reports confirm that pulmonary HAC can mirror HCC both radiologically and serologically,^13^ complicating preoperative differentiation. To resolve this diagnostic dilemma, systematic immunohistochemical profiling was performed on the BM specimen. The results yielded a clear phenotypic signature: strong and diffuse positivity for Arginase-1 (Arg-1) and Glypican-3 (GPC-3), against a background of consistently negative staining for pulmonary adenocarcinoma markers (TTF-1, Napsin A, and CK7). CK19 showed only weak and focal staining, falling below the threshold indicative of biliary differentiation.

This immunoprofile carries decisive diagnostic weight. The strong and diffuse Arg-1 expression represents a highly specific indicator of hepatocellular lineage, providing compelling evidence for HCC.^14–16^ Critically, the complete absence of CK7 expression—a marker that is diffusely positive in the vast majority of pulmonary HACs^17^—powerfully excludes this primary pulmonary mimic and strongly supports the diagnosis of HCC. This pivotal finding, coupled with the negative staining for TTF-1 and Napsin A, reliably rules out pulmonary adenocarcinoma and its hepatoid variants.^18^ Although GPC-3 positivity aligns with hepatocellular differentiation, its limited specificity diminishes its utility in discriminating HCC from HAC. The minimal CK19 expression effectively excludes cholangiocarcinoma and mixed hepatocellular cholangiocarcinoma, thereby strengthening the diagnosis of a pure hepatocellular lesion. Furthermore, the elevated Ki-67 proliferation index (∼60%) corresponds with the observed aggressive imaging phenotype and marked FDG avidity, together indicative of a high-grade malignancy.

The FDG hypermetabolism observed in the pulmonary lesion differs from the low-to-moderate uptake characteristic of most HCCs and overlaps with features of HAC. Nevertheless, this finding is consistent with established reports that poorly differentiated HCCs, those with extrahepatic spread, or certain molecular subtypes can exhibit marked FDG avidity,^19^ potentially due to heightened proliferative activity, GLUT-1 overexpression, and increased glycolytic flux.^20^ Given that the immunoprofile and serum AFP unequivocally support a hepatocellular origin, the FDG avidity in this case should be attributed to the inherently aggressive phenotype of this EHCC rather than to HAC, a pattern also reported in other aggressive HCCs.^21^

The clinical management of tumor stroke requires differentiation from conventional cerebrovascular accidents. Tumor-related hemorrhage often occurs in normotensive individuals and is more frequently associated with seizures. Owing to the rarity and poor prognosis of HCC brain metastases, no consensus exists regarding their treatment. Consequently, a personalized strategy is essential, based on a multidisciplinary assessment of the patient’s neurological status, overall tumor burden, hepatic functional reserve, and projected survival. In cases of hemorrhagic metastasis, surgical resection can aid in obtaining a histologic diagnosis, decompressing the brain, and potentially improving neurological function.

This study has several limitations. The foremost limitation is the inability to biopsy the pulmonary lesion due to family refusal during the patient’s acute neurological deterioration, which precluded histopathological confirmation of the primary site. Nevertheless, based on imaging findings, significantly elevated serum AFP, the characteristic immunohistochemical profile of the BM (Arg-1+/GPC-3+/TTF-1−/Napsin A−/CK19weak+), and the exclusion of other primary origins, we consider EHCC to be the most plausible diagnosis. Furthermore, as the clinical focus was on neurological rehabilitation rather than systemic antitumor therapy, the oncological prognosis could not be assessed.

## Conclusion

In managing patients with suspected tumor stroke, prompt neuroimaging is essential to identify hemorrhagic BM. When such metastases occur in the presence of a normal liver and a markedly elevated AFP level, EHCC should be strongly considered. Diagnosis relies on histopathological confirmation, optimally from surgical specimens, supported by immunohistochemistry to exclude mimics such as HAC. Although treatment options remain limited, a timely and multidisciplinary approach—integrating surgery where feasible—is crucial for optimizing neurological and oncological outcomes.

## Learning points

- Recognizing Tumor Stroke: Acute neurological deficits (e.g., sudden unconsciousness) warrant immediate neuroimaging (CT/MRI) to evaluate hemorrhagic metastasis. Key differentiators from cerebral stroke include the absence of hypertension and a higher frequency of seizure comorbidity.
- Diagnosing Ectopic HCC (EHCC): Suspect EHCC when liver imaging is normal yet an extrahepatic mass coexists with markedly elevated AFP (>2000 ng/mL). Intense FDG avidity on PET/CT supports the diagnosis, but histopathological confirmation remains essential.
- Therapeutic Principles: Surgery is first-line for resectable hemorrhagic metastases to prolong survival and improve quality of life. Management should be individualized based on: (1) Clinical status: neurological deficits, Child-Pugh class, and life expectancy and (2) Disease burden: AFP levels, extracranial disease control, and number of metastases. For patients with limited life expectancy, the focus should shift to metastasis stabilization and symptom relief.
- Multidisciplinary Coordination: The complex management of EHCC with brain metastasis and tumor stroke necessitates a structured, multidisciplinary approach involving neurosurgery, oncology, radiology, and pathology to guide diagnosis, intervention, and rehabilitation, while recognizing the impact of diagnostic and therapeutic limitations on prognostic evaluation.
